# Disentangling the syntrophic electron transfer mechanisms of *Candidatus* geobacter eutrophica through electrochemical stimulation and machine learning

**DOI:** 10.1038/s41598-021-94628-0

**Published:** 2021-07-23

**Authors:** Heyang Yuan, Xuehao Wang, Tzu-Yu Lin, Jinha Kim, Wen-Tso Liu

**Affiliations:** 1 Department of Civil and Environmental Engineering, University of Illinois, Urbana-Champaign, Urbana, IL 61801 USA; 2 Department of Civil and Environmental Engineering, Temple University, Philadelphia, PA 19122 USA

**Keywords:** Water microbiology, Environmental biotechnology

## Abstract

Interspecies hydrogen transfer (IHT) and direct interspecies electron transfer (DIET) are two syntrophy models for methanogenesis. Their relative importance in methanogenic environments is still unclear. Our recent discovery of a novel species *Candidatus* Geobacter eutrophica with the genetic potential of IHT and DIET may serve as a model species to address this knowledge gap. To experimentally demonstrate its DIET ability, we performed electrochemical enrichment of *Ca.* G. eutrophica-dominating communities under 0 and 0.4 V vs. Ag/AgCl based on the presumption that DIET and extracellular electron transfer (EET) share similar metabolic pathways. After three batches of enrichment, *Geobacter* OTU650, which was phylogenetically close to *Ca.* G. eutrophica, was outcompeted in the control but remained abundant and active under electrochemical stimulation, indicating *Ca.* G. eutrophica’s EET ability. The high-quality draft genome further showed high phylogenomic similarity with *Ca.* G. eutrophica, and the genes encoding outer membrane cytochromes and enzymes for hydrogen metabolism were actively expressed. A Bayesian network was trained with the genes encoding enzymes for alcohol metabolism, hydrogen metabolism, EET, and methanogenesis from dominant fermentative bacteria, *Geobacter*, and *Methanobacterium*. Methane production could not be accurately predicted when the genes for IHT were in silico knocked out, inferring its more important role in methanogenesis. The genomics-enabled machine learning modeling approach can provide predictive insights into the importance of IHT and DIET.

## Introduction

Anaerobic digestion is widely used to convert high-strength waste streams to biogas. This renewable energy source is composed of only ~ 60% methane (CH_4_) and needs downstream upgrade to increase the heating value^1^. Therefore, it is of practical significance to understand the metabolic pathways that drive biogas production and develop engineering strategies to increase CH_4_ content^2^. In anaerobic digestion, CH_4_ is produced predominantly via interspecies hydrogen transfer (IHT) and/or an acetoclastic pathway^3^. The former has been extensively studied as a classical model for syntrophy^4^, in which hydrogen-producing bacteria use proton as an electron sink for energy conservation, and hydrogenotrophic methanogens scavenge the produced hydrogen gas (H_2_) for methanogenesis^5^. Of the 155 methanogen cultures, 120 can use H_2_ as an electron donor, and 110 are strictly hydrogenotrophic^6^, underpinning the importance of IHT in many methanogenic environments.

Direct interspecies electron transfer (DIET) was recently discovered to be a novel syntrophic model for methane production with *Geobacter* acting as a key syntroph^7,8^. In an early study, the aggregates from an up-flow anaerobic sludge blanket reactor were characterized to be conductive and dominated by *Geobacter* spp.^7^ Adding conductive media into anaerobic digesters further accelerated methane production^9,10^. Because many *Geobacter* spp. were capable of extracellular electron transfer (EET)^11–13^, they were hypothesized to transfer electrons to methanogens through direct contact. This mechanism was later proven valid by co-culturing *Geobacter metallireducens* with *Methanosaeta harundinacea* or *Methanosarcina barkeri*^8,14^.

Model simulation suggests that electron transfer via IHT may be an order of magnitude slower than via DIET, and up to 33% of methane production can result from DIET^15,16^. However, the relative contribution of IHT and DIET to methane production remains inconclusive in both natural and engineered systems. A model species capable of both syntrophic electron transfer mechanisms is needed to address this knowledge gap.

The genus *Geobacter* is found dominant in many digester communities and may play a key role in methane production^17–21^. Multiple lines of evidence suggest that *Geobacter* spp. can form syntrophic associations with methanogens via IHT. For example, *Geobacter sulfurreducens* can oxidize acetate and produce H_2_ in the presence of hydrogen-oxidizing partners^22,23^. This species has been reported to produce H_2_ in bioelectrochemical systems^24^, leading to the enrichment of hydrogenotrophic methanogen^25^. From the *G. sulfurreducens* genome, four distinct [NiFe]-hydrogenase-encoding gene clusters that are putatively involved in hydrogen-dependent growth have been identified^26^.

It is reasonable to hypothesize that the *Geobacter* spp. dominant in methanogenic environments may transfer electrons simultaneously via IHT and DIET^27,28^, which allows them to be ecologically versatile and thrive under varying conditions. This hypothesis can be strengthened by our recent study of anaerobic reactors amended with conductive granular activated carbon (GAC), where we observed high abundances of *Geobacter*-related taxa (up to 55%) and enhanced methane production^20^. The most dominant population was confirmed to be a novel *Geobacter* species (*Candidatus* Geobacter eutrophica strain bin GAC1) based on the phylogenetic and phylogenomic analyses. Its draft genome revealed the genetic potential of both IHT and DIET, including the *ech* cluster for energy-conserving [NiFe] hydrogenase complex^29^, a cytoplasmic bidirectional NAD-reducing HoxPLSUFE complex, a periplasmically oriented membrane-bound HyaSLBP complex^26^, and 38 protein-coding sequences for c-type cytochromes that were putatively involved in EET^30^.

The objective of this study is to experimentally confirm *Ca.* G. eutrophica’s DIET ability through electrochemical stimulation based on the working hypothesis that it uses similar metabolic pathways for DIET and EET^12,31^. If *Ca.* G. eutrophica performs DIET, it may also use an anode electrode as an electron acceptor and can be electrochemically stimulated and enriched. To this end, *Ca.* G. eutrophica-dominating GAC was collected from our previous study and enriched in two-chamber bioelectrochemical systems with the electrode poised at positive potential^20^. After three batches of enrichment, we sequenced the 16S rRNA and rRNA gene to characterize the overall microbial activity and community structure, respectively. We also performed metagenomic and metatranscriptomic analyses to understand the ecophysiology of the dominant *Geobacter* and their fermentative and methanogenic partners. Finally, we developed a Bayesian network approach to provide predictive insights into the importance of IHT and DIET to methanogenesis.

## Results and discussion

### Effects of enrichment on system performance

**Figure 1:**
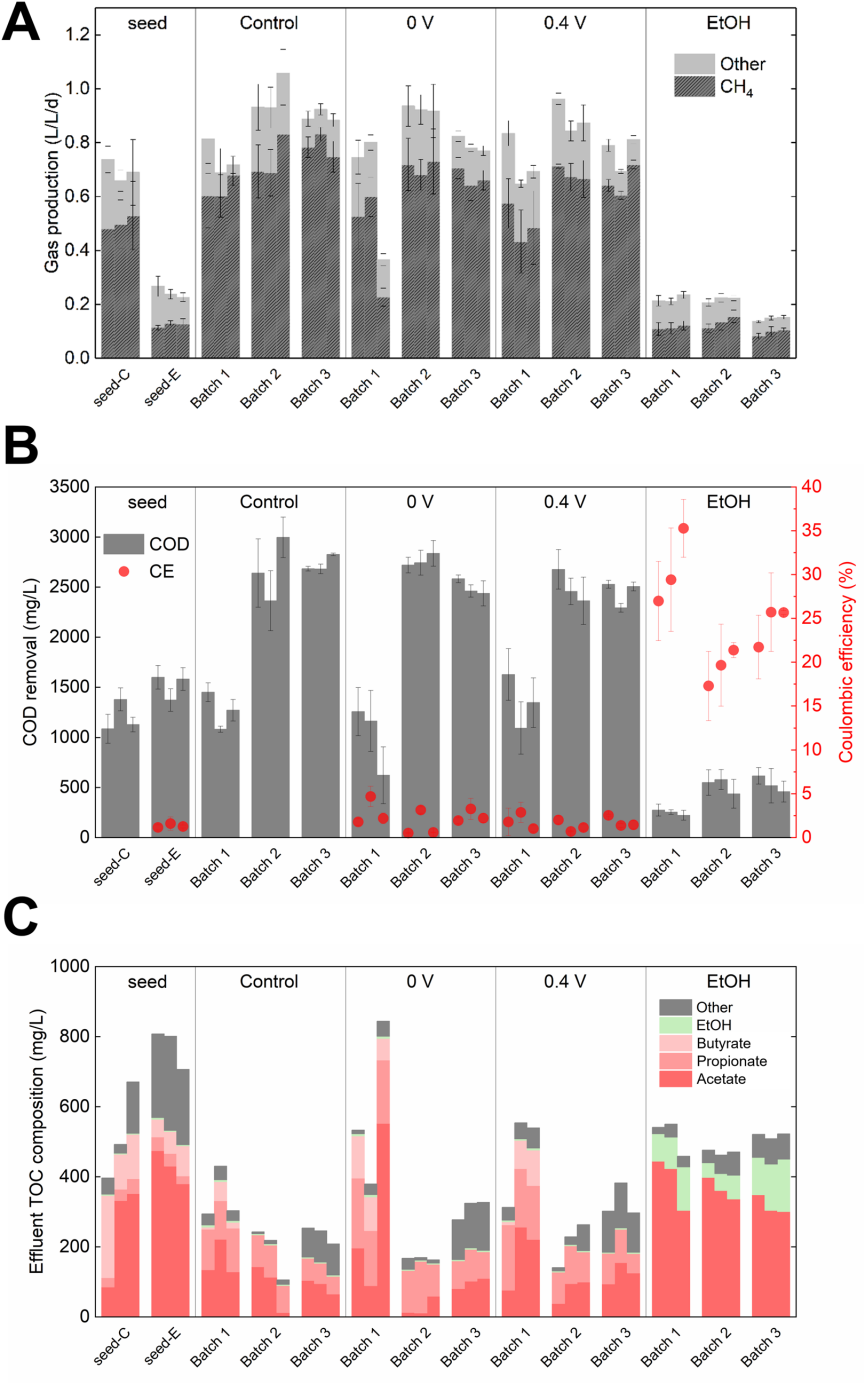
(**A**) Biogas production, (**B**) COD removal and CE, and (**C**) effluent composition under different potential and substrate conditions. Seed-C and seed-E were the control and electrochemical seeds, respectively. Control, 0-V, and 0.4-V reactors were fed with fructose and PEG. EtOH reactors were fed with ethanol.

Before enrichment, a cultivation step was performed to alleviate potential electrochemical shock to the methanogenic population by transferring *Ca.* G. eutrophica-containing GAC from a packed-bed bioreactor to bioelectrochemical systems amended with fresh GAC. After 20 cycles (60 days) of cultivation, the electrochemical seed (0 V vs. Ag/AgCl) showed lower methane production than the control (0.1 L/L/d vs. 0.5 L/L/d, Fig. 1A) but similar chemical oxygen demand (COD) removal of about 1200 mg/L (Fig. 1B). Additionally, acetate accumulation was observed in the electrochemical seed effluent (up to 50% of the total organic carbon (TOC), Fig. 1C). The results indicate strong electrochemical shock to the methanogenic population, particularly acetoclastic methanogens, during the initial cultivation. GAC was collected from the seeds for enrichment (Figure S1).

In Batch 1, the methane production rate of the control reactors increased slightly to 0.6 L/L/d (Fig. 1A), while that of the 0-V reactors increased significantly to 0.4 L/L/d, suggesting recovery of the methanogenic population from electrochemical shock. One of the 0-V reactors produced low biogas throughout the first batch due to rapid pH drop (average 5.2 after each cycle, Figure S2), but the reason was not clear. The 0-V reactors exhibited more stable performance in the following batches and produced CH_4_ at a rate of 0.6 L/L/d. Higher potential (0.4 V vs. Ag/AgCl) did not affect methane production, and both 0-V and 0.4-V reactors produced CH_4_ slightly slower than the control (p < 0.05). Another group of reactors were operated at 0 V but fed with ethanol as the sole carbon source, as described in pure-culture DIET studies^8^. However, the EtOH reactors produced CH_4_ at a much lower rate of 0.1 L/L/d in all batches.

At the early stage of the enrichment, the reactors fed with fructose and polyethylene glycol (PEG) showed similar COD removal of about 1200 mg/L, whereas the EtOH reactors removed only 250 mg/L COD (Fig. 1B). COD removal doubled for all reactors in the following batches, but this enhancement was not reflected by CH_4_ production, which increased by < 20% (Fig. 1A). In the meantime, coulombic efficiency (CE) showed a decreasing trend and dropped by ~ 3% for the fructose/PEG-fed electrochemical reactors and by 10% for the EtOH reactors. High COD removal and low CE are expected as a significant proportion of the electrons will be channeled to biomass, metabolites, and CH_4_ when fermentable substrates are fed as the electron donor^32^.

The concentrations of short-chain volatile fatty acids (VFAs, including acetate, propionate, and butyrate) and ethanol in the effluent were converted to TOC for comparison (Fig. 1C). For the electrochemical reactors, effluent TOC dropped from 800 mg/L in the seeds to about 500 mg/L in Batch 1. The abnormally high TOC found in one of the 0-V reactors in this batch was consistent with its gas production and COD removal and was attributed to the low pH that caused reactor failure. Total VFAs in those reactors further decreased to below 200 mg-TOC/L in Batch 2 and 3 with noticeable propionate accumulation. Ethanol was detected with a low concentration (< 10 mg-TOC/L), which was not in agreement with our previous study^20^. For the EtOH reactors, effluent TOC remained stable throughout the enrichment and consisted of 400 mg-TOC/L acetate followed by 100 mg-TOC/L ethanol. High acetate concentration in the effluent and low CH_4_ production together suggest inhibition of acetoclastic methanogenesis, likely because the presence of acetate and electrode favors the growth of electroactive acetate scavengers and potentially leads to the washout of acetoclastic methanogens^33^.

### Effects of enrichment on microbial population and overall activity

A clear shift in the community structure in the 0-V and 0.4-V reactors can be seen from the 16S rRNA gene-based principal coordinate analysis (PCoA) results (Figure S3). The communities in those reactors became significantly different from the seed communities (permutational multivariate analysis of variance (PERMANOVA), p < 0.05) and clustered closely with the control communities in Batch 3, demonstrating successful enrichment. Similar results were reported by a previous study, in which poised potentials exerted minor impacts on *Geobacter*-dominating microbial communities^34^. The EtOH-fed communities, on the other hand, were separated from the fructose/PEG-fed communities since Batch 1 and remained stable throughout the enrichment. The significant difference in microbial communities agreed well with the system performance (Fig. 1), highlighting the key role of the substrate as a deterministic factor that drives microbial community dynamics^35,36^.

**Figure 2:**
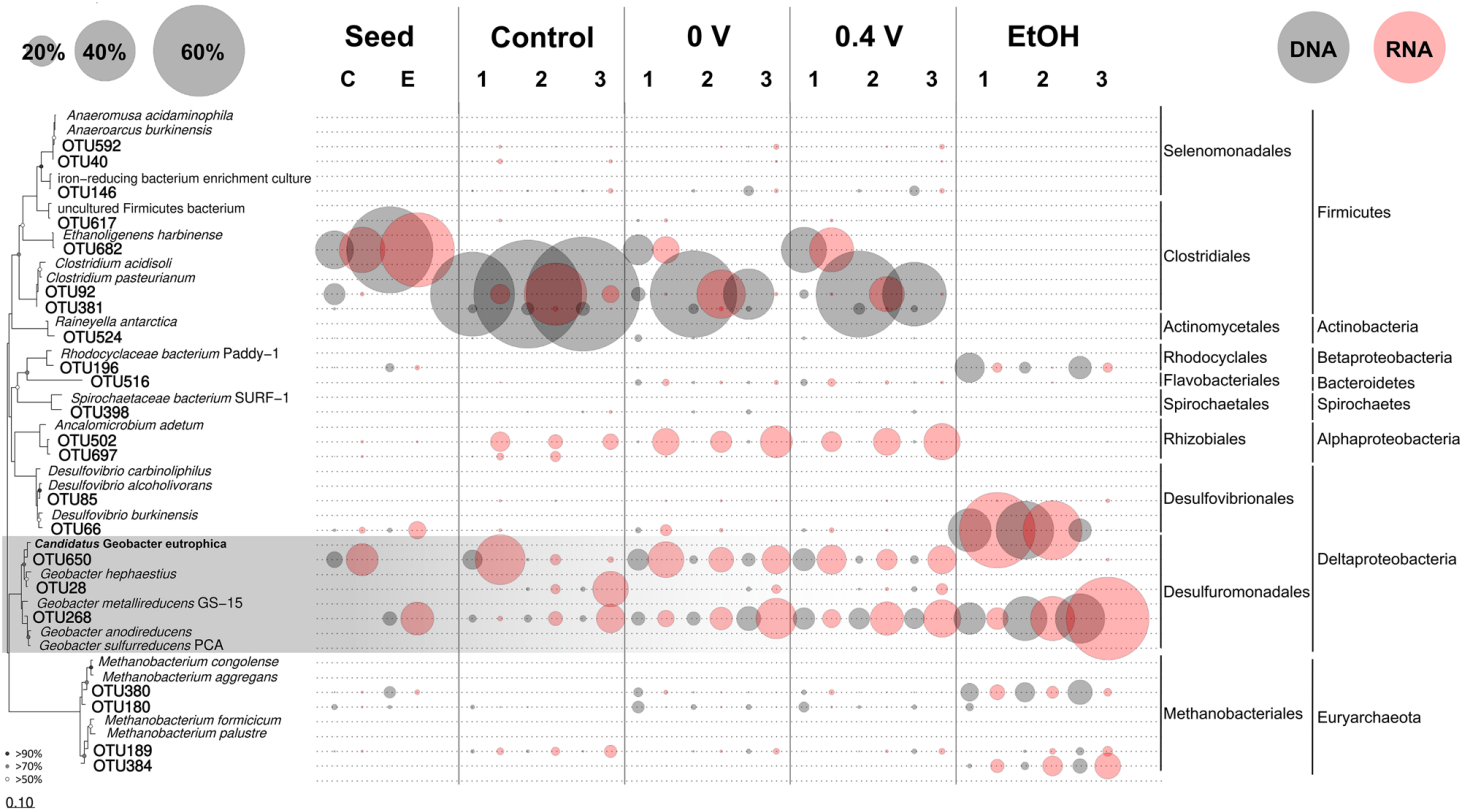
Phylogenic tree, relative abundance (16S rRNA gene), and activity (16S rRNA) of 22 core OTUs under different potential and substrate conditions.

A core population composed of 22 operational taxonomic units (OTUs) was selected based on the criteria of average abundance > 0.5% and occurrence > 50% across all DNA and RNA samples (Fig. 2). In the 0-V and 0.4-V reactors, *Ethanoligenens* OTU682 was initially abundant (57% 16S rRNA gene abundance) and active (49% 16S rRNA abundance) and was replaced by *Clostridium* OTU92 during the enrichment. *Rhodocyclaceae* OTU196 and *Desulfovibrio* OTU66 were the dominant taxa in the EtOH reactors. They are potentially primary fermentative bacteria that provide substrates for electroactive bacteria and methanogens^37–39^. *G. sulfurreducens*-related OTU268 was stimulated and enriched in all electrochemical reactors, and the correlation with CE (Figure S4) revealed its electroactive nature^40^. Also enriched were strict hydrogenotrophic *Methanobacterium* spp^41,42^.

*Geobacter* OTU650 is of particular interest due to its high phylogenetic similarity to *Ca*. G. eutrophica (Fig. 2). It was outcompeted in the control but still present under electrochemical stimulation. Although its abundance decreased to between 5%—10% in the 0-V and 0.4-V reactors in Batch 3, OTU650 was still among the top three most abundant taxa. Moreover, it contributed to 15% to 20% of the community 16S rRNA and showed high activity throughout the enrichment. OTU650 was absent in the EtOH reactors and might not be a competitive utilizer of ethanol and acetate while respiring on an electrode. Instead, Activity-based redundancy analysis (RDA) predicted the association between this taxon and propionate (Figure S4). OTU650 is also predicted by RDA to be involved in CH_4_ production. The significant difference between the control and electrochemical reactors (p < 0.05) confirms that *Ca*. G. eutrophica-related OTU650 is capable of EET. Meanwhile, the minor difference under different potentials implies the presence of upper stream rate-limiting factors such as the availability of propionate (as the electron donor), which is determined by the metabolic activity of the fermentative partners.

### Fermentative bacteria providing substrate for methanogenesis

**Figure 3:**
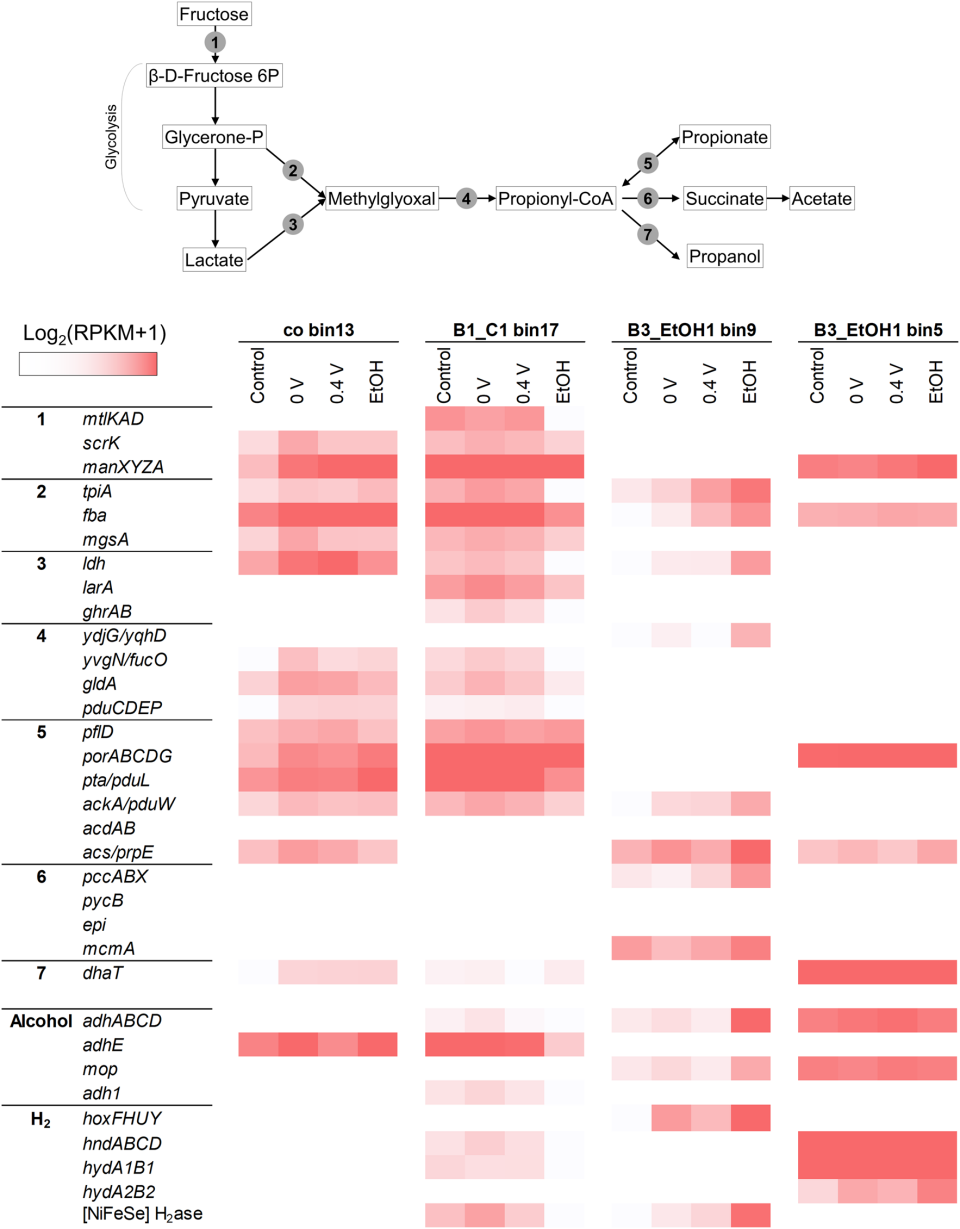
Metabolic reconstruction for the four dominant fermentative bacteria. Heatmap is created using Microsoft Excel (Microsoft 365).

Metagenomic analysis yielded 28 high-quality metagenome-assemble genomes (MAGs) with > 98% completeness and < 2% contamination (Figure S5), whose bin coverage and percentage of mapped reads in Batches 1 and 3 agreed well with the relative abundance of the selected core population shown in Fig. 2. We reconstructed the metabolic pathways for fructose utilization, propionate/propanol accumulation, ethanol production/utilization, and H_2_ metabolism for the four primary fermentative bacteria (Fig. 3).

*Ethanoligenens* co_bin 13 was abundant in the 0-V and 0.4-V reactors in Batch 1 (Figure S5). The genes for fructokinase (*scrK*), mannose phosphotransferase (*manXZ*), and mannose isomerase (*manA*) were highly expressed, suggesting that co_bin 13 could metabolize fructose directly or with mannose as an intermediate. The resulting β-D-fructose 6-phosphate was fed into glycolysis and led to the accumulation of propionate through the generation of glycerone phosphate, methylglyoxal, and propionyl-CoA (Fig. 3). It actively expressed six genes that are important for the conversion of propionate/propionyl-CoA, but the genes for downstream propionyl-CoA utilization were not detected. Similar to other *Ethanoligenens* species^43–45^, co_bin 13 could carry out complete glycolysis to produce ethanol, as indicated by the expression of the *adhE* gene encoding aldehyde-alcohol dehydrogenase. However, genes for H_2_-evolving hydrogenase were not found in its draft genome. The results collectively suggest that *Ethanoligenens* co_bin 13 ferments fructose to ethanol, propionate, and propanol.

*Clostridium* B1_C1_bin17 was ubiquitously abundant in the fructose-fed reactors (Figure S5). In addition to the two fructose metabolism pathways mentioned above, *Clostridium* B1_C1_bin17 expressed a third route with mannitol as an intermediate. It could convert the produced β-D-fructose 6-phosphate to propionate-CoA either via the glycerone phosphate-methylglyoxal route or through lactate metabolism (Fig. 3), further underpinning its metabolic flexibility. B1_C1_bin17 could also be a major propionate producer. The ability to produce 1,3-propanediol and propanol by *Clostridium* species was observed in B1_C1_bin17 through the expression of the *dhaT* gene^38,46^, a 1,3-propanediol dehydrogenase-like enzyme that potentially catalyzed NADH-dependent propanal reduction to propanol. Finally, B1_C1_bin17 metabolized ethanol by expressing three alcohol dehydrogenase-encoding genes and the genes for the periplasmic [NiFe] and [NiFeSe] hydrogenases. The expression was one order of magnitude higher in the fructose-fed reactors than in the EtOH reactors, suggesting that it could couple fructose degradation to ethanol metabolism with proton as an electron sink. Overall, *Clostridium* B1_C1_bin17 contributes propionate, propanol, and H_2_ to methanogenesis.

*Rhodocyclaceae* B3_EtOH1_bin9 and *Desulfovibrio* B3_EtOH1_bin5 preferred ethanol as the substrate (Figure S5). Genomic analyses confirmed that the *Rhodocyclaceae* population was incapable of fructose metabolism, and both were deficient in propionate production (Fig. 3). The *Rhodocyclaceae* population showed the ability to metabolize ethanol and carried the genes for the NAD-reducing Hox complex and a periplasmic [NiFeSe] hydrogenase, which indicated its role as a major H_2_-donating partner. The *Desulfovibrio* population also actively expressed the genes for periplasmic [Fe] and [NiFe] hydrogenase complexes that allowed it to use proton as an electron acceptor and grow syntrophically with H_2_-scavenging partners^47,48^. Alternatively, under electrochemical stimulation, *Desulfovibrio* B3_EtOH1_bin5 may use its own H_2_ as an electron donor to respire on the poised electrode. B3_EtOH1_bin5 showed high activity of the Hnd complex that was recently reported to transfer electrons from H_2_ to NADH through flavin-based bifurcation^49^. We also observed high activity of the menaquinone reductase complex (Qrc) involved in sulfate respiration^50^, which might explain the EET ability found in several *Desulfovibrio* spp.^51,52^.

### *Geobacter* playing a key role in methanogenesis

**Figure 4:**
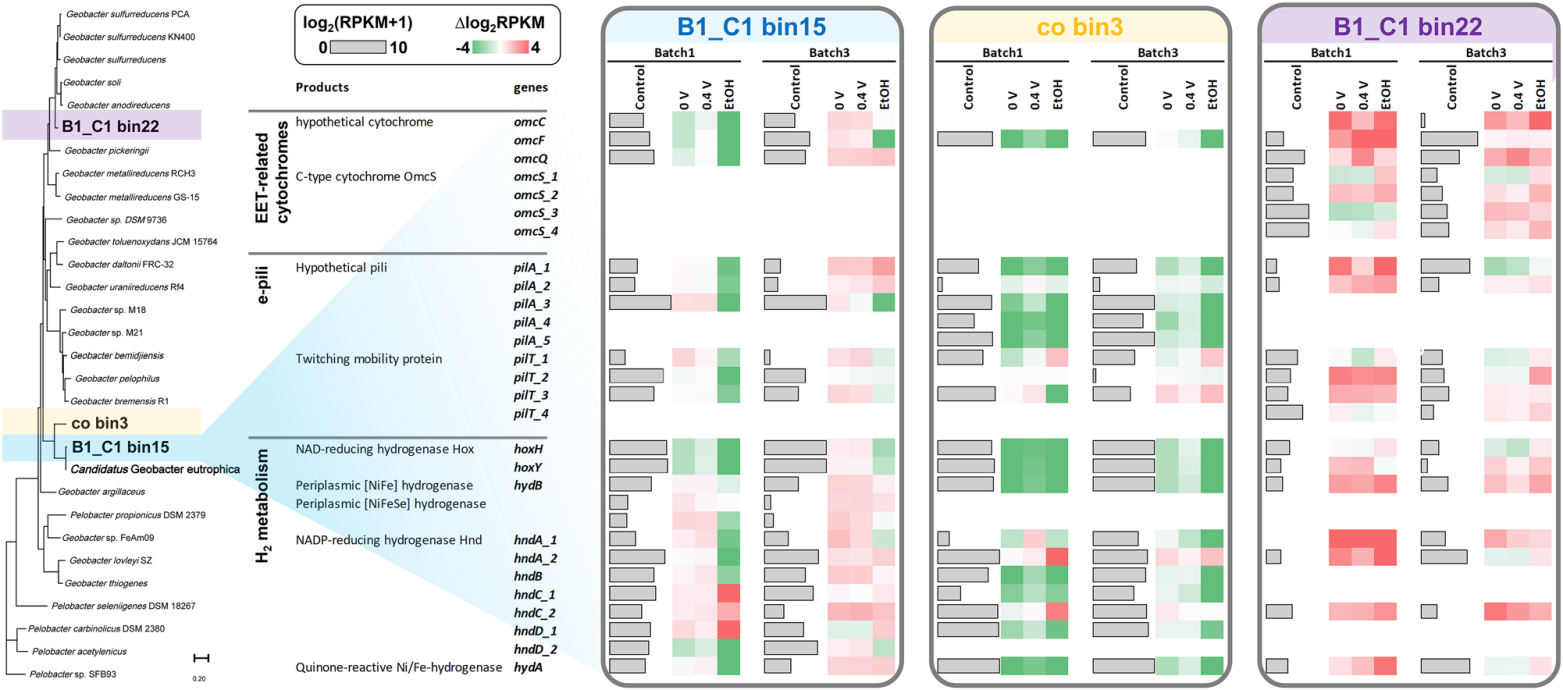
Expression of genes encoding outer membrane c-type cytochromes, conductive pili, and H_2_-evolving/-uptake hydrogenases of three abundant *Geobacter* spp. in Batch 1 and 3. The gene expression levels in the control reactors are expressed as log_2_(RPKM + 1), while those in the electrochemical reactors are expressed as the relative values to the control. Heatmap is created using Microsoft Excel (Microsoft 365).

*Geobacter* B1_C1_bin15 (OTU650) was recovered with an almost complete genome (completeness > 99.4% and contamination < 0.6%) and confirmed to be phylogenomically nearly identical to *Ca.* G. eutrophica (Fig. 4). Metabolic construction reveals a complete set of genes for propionate metabolism, seven copies of the *dhaT* gene for 1,3-propanediol dehydrogenase for propanol metabolism, and high activity of several types of alcohol and aldehyde dehydrogenases (Figure S6). These results are consistent with the previous finding that *G. metallireducens* grows syntrophically with DIET partners on propionate, propanol, and ethanol^17^. However, the absence of *Ca*. G. eutrophica-related OTU650 in the EtOH reactors (Fig. 2) suggests that it prefers carbon sources other than ethanol when carrying out EET/DIET.

One of the potential carbon sources is PEG. It was seen from the metatranscriptomic analysis that the genes for aldehyde dehydrogenase (*aldHT*) and oxidoreductase (*mop*) are significantly higher in the fructose/PEG-fed reactors than in the ethanol-fed reactors (p < 0.05, Figure S6). Based on the difference, we speculated that those enzymes and long-chain-alcohol dehydrogenase are putatively involved in PEG degradation^53^. Anaerobic degradation of PEG has long been observed in *Pelobacter* spp.^54^ a member from the order of Desulfuromonadales to which *Geobacter* also belongs. Previous studies have consistently detected propionate as the byproduct during PEG degradation^55,56^, and the produced propionate can be further metabolized by *Ca*. G. eutrophica (Figure S6). Whether *Ca*. G. eutrophica can degrade PEG and couple PEG/propionate degradation to DIET warrants further inspection.

Through mapping the metatranscriptomic reads to a manually curated database, we identified three outer membrane c-type cytochromes that were slightly more active (up to 2 folds, p < 0.05) in the electrochemical reactors after enrichment (Fig. 4). Among them, OmcF appears to be required for the transcription of the gene encoding OmcC that is directly responsible for Fe(III) reduction and potentially GAC-mediated EET/DIET in this study^57^. Three copies of the conductive pili-encoding *pilA* gene were also detected but did not show a noticeable difference under different conditions. One of the *pilA* genes, with 72% identity and 100% coverage relative to that found in *G. metallireducens*, was actively expressed, implying its involvement in EET/DIET.

We also observed the expression of the genes for putative H_2_-evolving hydrogenases (Fig. 4). The Hox complex was globally active across batches and conditions, while the periplasmic [NiFe] and [NiFeSe] complexes became slightly less active after enrichment but still showed twofold higher expression under electrochemical stimulation. Further inspection of the B1_C1_bin15 genome revealed that it carries a quinone-reactive Ni/Fe hydrogenase and an Hnd complex in which the genes (*hndABCD*) are 55–81% similar (> 98% coverage) to those found in *Desulfovibrio fructosovorans*. Both were highly active in the electrochemical reactors and could be involved in H_2_ uptake couple with EET to electrode^49^.

*Geobacter* co_bin3 is phylogenomically close to *Ca.* G. eutrophica (Fig. 4) and seems to compete with *Geobacter* B1_C1_bin15 for the same ecological niche based on their interactive changes in abundance (Fig. 2 and Figure S5). This is supported by the gene expression profile, which shows lower activity of the *omcF* gene, five copies of the *pilA* gene, and the genes for H_2_-evolving hydrogenases and the Hnd complex under electrochemical stimulation.

Unlike the other two members in this genus, *Geobacter* B1_C1_bin22 is ubiquitously abundant in both fructose- and ethanol-fed electrochemical reactors. Its phylogenomic similarity to *G. sulfurreducens* is reflected by the carriage of a number of cytochromes from the Omc family (Fig. 4), including four copies of the well-characterized OmcS that is highly upregulated during EET to Fe(III) oxide and electrode^11^. This population also showed activity of both H_2_-evolving and H_2_-uptake hydrogenases, with the latter potentially playing a central role in its growth. Similar to *G. sulfurreducens*, B1_C1_bin22 is not likely to grow on ethanol^58^. It expressed only one gene for putative alcohol dehydrogenase and lacked acetaldehyde dehydrogenase. Therefore, *Geobacter* B1_C1_bin22 may compete with hydrogenotrophic methanogens on H_2_ as an electron donor for EET, thereby suppressing methane production and producing a high CE in the EtOH reactors (Fig. 1).

### Hydrogenotrophic methanogens with DIET potential

Although the bioelectrochemical systems were designed to stimulate DIET-capable *Geobacter*, we observed high abundances of several *Methanobacterium* spp. with high phylogenomic similarity to known species (Figure S5) and retrieved near-complete genomes (> 99% completeness and < 1% contamination). These strict hydrogenotrophic methanogens use three routes to recycle coenzyme M and coenzyme B at the end of the methanogenic pathway (Figure S7). The ferredoxin:CoB-CoM heterodisulfide reductase (HdrA1B1C1), a homolog of the HdrABC complex commonly found in most methanogens, became less active in the control. On the other hand, they possess the heterodisulfide reductase [NiFe]–hydrogenase complex for flavin-based electron bifurcation coupled with ferredoxin reduction and H_2_ oxidation^59^, and the genes encoding the complex (*hdrABC/mvhADG*) were highly active in all treatments. Membrane-bound heterodisulfide reductase (*hdrD*) and F420 dehydrogenase (*fpoD*), which were proposed to participate in extracellular electron uptake^6^, were also present but not active after enrichment, indicating their weak roles in methane production. An interesting finding is that the *Methanobacterium* spp. carry up to seven copies of the *mvhB* gene that are actively expressed across different batches and conditions, and the encoded polyferredoxins with high iron content have been speculated to participate in electron transfer^60,61^. It is possible that the *Methanobacterium* spp. use polyferredoxins to shuttle extracellular electrons to the MvhADG/HdrABC complex to complete DIET, but the specific electron uptake and transfer mechanisms need further investigations.

### A predictive understanding of the methanogenic population and electron transfer mechanisms

Bayesian network analysis was performed to predict the microbial interactions and the potential functions of key taxa in a given microbial ecosystem^62^. To investigate the effects of the input data type on network training, we selected 17 and 20 OTUs from the DNA and RNA datasets (abundance > 0.5% and occurrence > 50% in each dataset), respectively. The modeling method was first validated by reconstructing the core populations. The Bray–Curtis similarities between the predicted and observed communities (0.62 for DNA and 0.64 for RNA) were significantly higher than those from a null model (Figure S8). Although the predictive power might be compromised by functionally redundant taxa at a high taxonomic resolution^62^, the simulation was more accurate than that yielded by artificial neural networks (constructed to predict acid mine drainage communities)^63^. The Bayesian network approach was further validated by correlating the predicted and observed system performance (Table S1). Satisfactory predictions were achieved with an average R^2^ > 0.61 and root-mean square error (RMSE) of 0.11. Among the parameters examined, methane production was predicted with the highest accuracy, and the R^2^ with the RNA dataset reached 0.94. Other parameters such as acetate and ethanol in the effluent were also better predicted with RNA than with DNA. Overall, RNA was a more robust indicator than DNA with a significantly higher average R^2^ (0.69 vs. 0.61) and lower RMSE (p < 0.05), demonstrating the strong connection between microbial activity and system performance.

**Figure 5:**
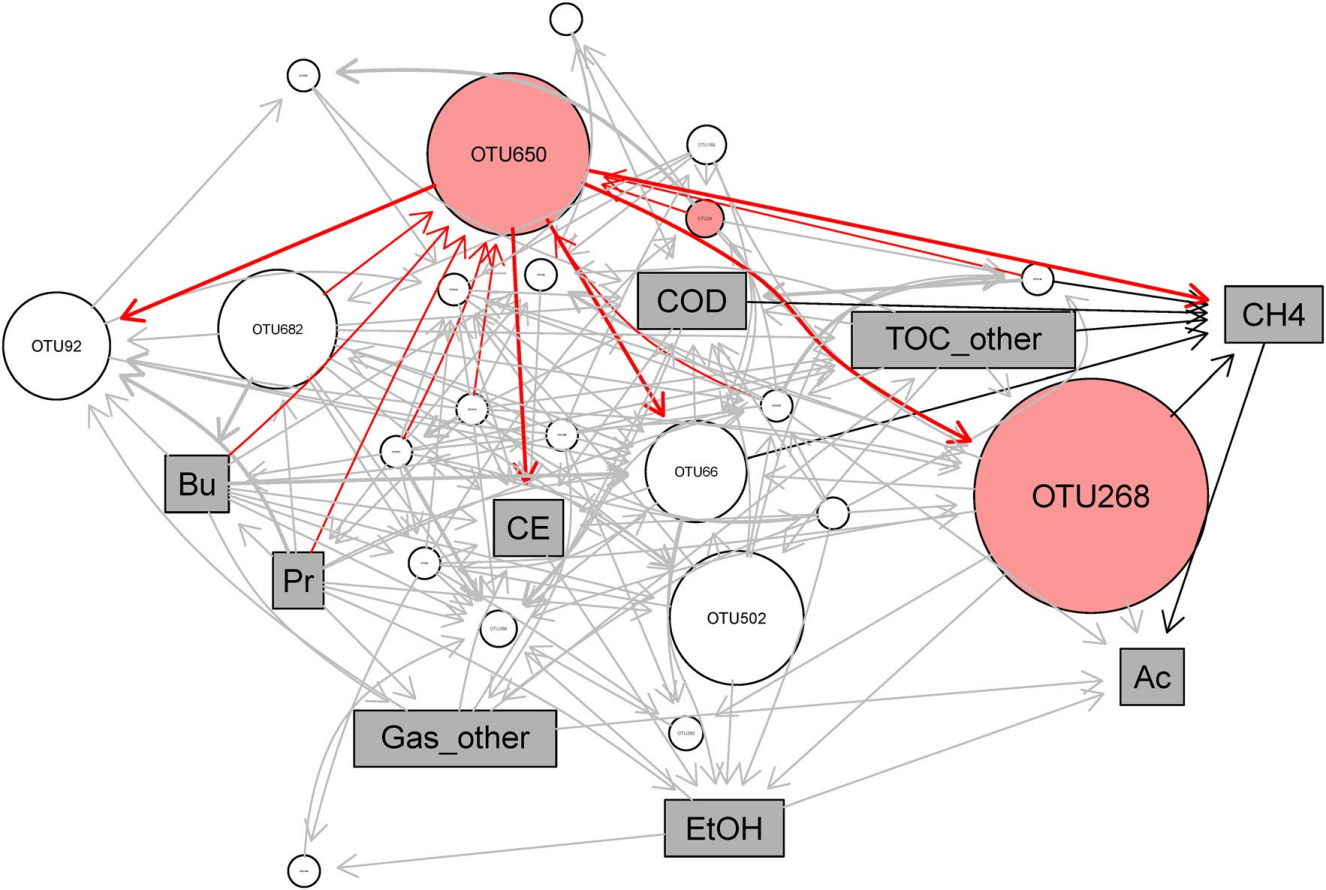
Bayesian network constructed with the activity (16S rRNA) of the 22 core OTUs (round nodes) and 9 environmental parameters (rectangular nodes). *Geobacter* spp. and their interactions with other nodes are highlighted in red. Networks are visualized using Rgraphviz (2.36.0)^95^.

After the Bayesian network modeling approach was validated with Bray–Curtis similarities and system performance predictions, a final network was constructed with the RNA dataset at the OTU level (Fig. 5). The inference direction from propionate to *Ca*. G. eutrophica-related OTU650 implies its potential role as a propionate utilizer, which is consistent with the results from RDA (Figure S3) and metabolic reconstruction (Figure S6). All three *Geobacter* taxa in the core population were associated with methane production. OTU650 was assigned with the highest positive coefficient (0.42) followed by OTU28 (0.36), confirming their contribution to methanogenesis via IHT and/or DIET (Fig. 4). OTU268 showed a negative association (coefficient -0.12) and potentially competed with hydrogenotrophic methanogens on H_2_. As revealed by the metatranscriptomic profiling, this *G. sulfurreducens-*related taxon is incapable of complete oxidation of ethanol but actively expressed hydrogenases for H_2_ uptake (Fig. 4). The inference could also explain the less accurate prediction of methane production at a higher taxonomic level (i.e., at the genus level, Table S1). In the genus-level network, the three physiologically distinct *Geobacter* OTUs were combined under the same genus, and their individual impacts on methanogenesis were neutralized, leading to a decrease in the predictive power. The inference presents a significant step toward interpreting black-box machine learning models by providing appropriate inputs (i.e., 16S rRNA) for model training^64^.

**Figure 6:**
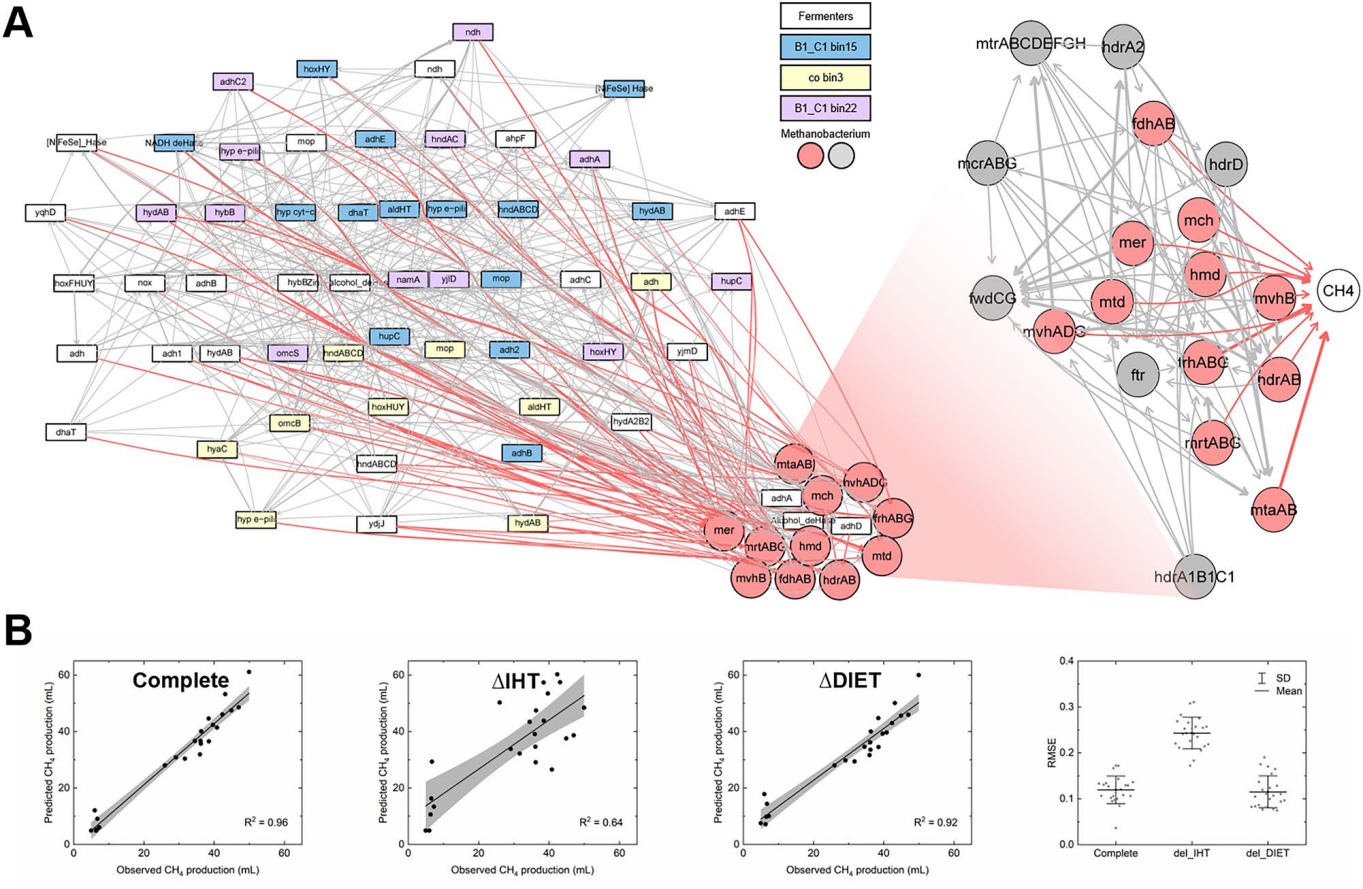
(**A**) Bayesian network constructed with genes for proteins related to ethanol/propanol metabolism, EET, H_2_ metabolism, and methanogenesis. The genes were extracted from four fermentative bacteria, three *Geobacter*, and three *Methanobacterium* spp. (**B**) Correlation between predicted and observed methane production and RMSE with all genes (complete), genes for H2 metabolism silenced (∆IHT), and genes for EET silenced (∆DIET). Networks are visualized using Rgraphviz (2.36.0)^95^.

We applied the Bayesian modeling approach to the metatranscriptomic data to gain a predictive insight into the importance of IHT and DIET to methanogenesis. Using the MAGs of the four fermentative bacteria, three *Geobacter* spp., and three methanogens discussed above, we extracted the genes related to alcohol oxidation (both ethanol and propanol), H_2_ metabolism (both H_2_ evolution and uptake), and the putative genes for DIET (e.g., the *omc* genes in *Geobacter* and the *mvhB* genes in *Methanobacterium*) to train the model (Fig. 6A). The Bayesian network is composed of two components: upstream gene–gene interactions for predicting the expression level of the relevant genes in the methanogens, and a downstream sub-network that links the methanogen genes to methanogenesis. Such a network structure allows us to impose the inference direction strictly from primary/secondary fermentative bacteria over methanogens to methane production, thereby reconstructing the metabolic interactions among the key partners. A complete network could yield a highly accurate prediction of methane production with R^2^ = 0.96 and RMSE = 0.11 (Fig. 6B). To statistically infer the importance of DIET and IHT, we manually silenced relevant genes. The prediction power without the IHT-related genes was significantly compromised (Fig. 6B), as evidenced by a lower R^2^ value of 0.64 and a higher RMSE of 0.24. Manually setting the expression level of the DIET-related genes to zero slightly decreased the R^2^ value of 0.92. This *in-silico* knockout strategy thus implies that IHT plays a more critical role than DIET in methane production in the electrochemical reactors.

IHT is a ubiquitous electron transfer mechanism found in many methanogenic environments^6^. On the other hand, DIET was believed to be the dominant mechanism in bioreactors amended with conductive media^21^. Model simulation suggests that DIET is faster than mediated electron transfer and can contribute to up to 33% of the produced methane^15,16^. However, the relative importance of IHT and DIET is still a much debated topic due to the technical difficulty to experimentally disentangle those two electron transfer mechanisms. *Ca*. G. eutrophica capable of IHT and DIET/EET can serve as a model species for understanding the effects of environmental factors on electron transfer. From an engineering perspective, bioreactors enriched with this metabolically versatile species may be more resilient to operational fluctuations. By far, *Ca*. G. eutrophica has not been found in natural ecosystems, and their ecological implication warrants further investigation.

## Methods

### Reactor setup and operation

Two-chamber bioelectrochemical systems were built as previously described^65^. The anode (total volume 130 mL) was amended with 20 mL of fresh GAC and inoculated with 5 mL of cultivated GAC. The inoculum was collected from an up-flow packed-bed bioreactor that had been operated for three months according to our previous study^20^, which resulted in a high abundance of *Ca.* G. eutrophica. The anode electrode was a stainless-steel brush immersed in GAC. An Ag/AgCl electrode [3 M KCl, 0.21 V vs. SHE (standard hydrogen electrode)] was installed adjacent to the anode electrode as a reference. The reactors were fed with 100 mL of substrate mimicking soft drink-processing wastewater^19^. The substrate contained 1500 mg/L fructose and 1100 mg/L PEG 200 as the carbon sources and was slightly modified by adding 50 mM phosphate buffer saline to buffer the pH change. The influent COD was 3000 mg/L. The catholyte was 100 mM phosphate buffer saline. The reactors were operated under a batch mode with a hydraulic retention time of three days.

To prepare seeds for enrichment, the anode potential was poised at 0 V vs. Ag/AgCl using a multi-channel potentiostat (MPG-2, BioLogic). This potential is more positive than the typical redox potentials of *Geobacter*’s c-type cytochromes (-200 mV vs. SHE)^66,67^, allowing the anode electrode to act as an electron sink. The control reactors were operated under an open-circuit condition. Enrichment started after 60 days of incubation (20 cycles), and 5 mL GAC was collected to seed Batch 1. The electrochemically stimulated reactors were further operated under three conditions: 0 V, 0.4 V, and 0 V + ethanol. The COD of the ethanol-based substrate was adjusted to 3000 mg/L. The enrichment process was repeated three times, and each lasted 20 cycles (60 days) to ensure the establishment of a stable microbial community. All reactors were operated in triplicate at 37 $^\circ$C. A schematic of the enrichment process is shown in Supporting Information (SI) Figure S1.

### Chemical analysis

Samples were collected in the last three cycles of each batch. Biogas produced by the reactors was collected using water displacement for volume and rate measurement. After moisture removal, CH_4_ concentration was determined using a GC-2014 Gas Chromatograph (Shimadzu) equipped with a Molecular Sieve 13X packed column (2000 × 2 mm, Restek) and a thermal conductivity detector. For chemical measurements, effluent samples were filtered through 0.22 µm membranes. Soluble COD was measured with a COD digestion kit and DR/4000 U Spectrophotometer (HACH). TOC was measured with a TOC-V Analyzer (Shimadzu). VFAs were measured with a 1200 Series HPLC System equipped with a Hi-Plex H column for organic acids (Agilent). Ethanol concentration was measured with a GC-2014 Gas Chromatograph (Shimadzu) equipped with a flame ionization detector. The pH of the effluent was measured using a benchtop pH meter (Fisher). Current production resulted from poised potential was recorded with the manufacturer's software (BioLogic), and CE was calculated as previously described^68^.

### Nucleic acid extraction and sequencing

Triplicate GAC samples were collected at the end of each batch and stored immediately at -80 °C before nucleic acid extraction. DNA extraction, library preparation, amplicon sequencing, and microbial community analyses were performed as previously described^36,62^. RNA was extracted following the manufacture’s instruction (Qiagen) and purified with both RNase-free DNase Set (Qiagen) and Turbo DNA-free (Ambion). Genomic DNA in the purified RNA was not detected using DNA-targeting PCR, and the treated products were reverse transcribed to cDNA using a random hexamer (Invitrogen). The V4-V5 region of the 16S rRNA gene was amplified with the prokaryote universal primer pair 515F/909R. Purified PCR products were pooled in equalmole ratios and sequenced on an Illumina Miseq Bulk 2 × 300 bp paired-end platform. One reactor under each operating condition was collected from Batches 1 and 3 for metagenomic sequencing (8 samples), and all three reactors were collected from Batches 1 and 3 for metatranscriptomic sequencing (24 samples). Sequencing was performed on an Illumina NovaSeq 6000 platform to generate 2 × 250 bp paired-reads.

### Community analysis

Sequencing data are available in GenBank under the accession number PRJNA689312. Paired-end sequences were assembled and denoised using QIIME 2, and OTUs were picked using DADA2^69,70^. Taxonomy was assigned using the QIIME 2 plugin feature-classifier and the Greengenes database as the reference, with the classifier trained on 97% OTUs^71,72^. Statistical analyses, including two-sample t-test, linear regression, PCoA (based on weighted UniFrac distance)^73^, and RDA, were carried out using R. PCoA and RDA were performed on relative abundance (16S rRNA gene) and microbial activity (16S rRNA), respectively. PERMANOVA was carried out to test for the significant difference of PCoA results (N = 999). The relationships between environmental parameters and taxonomic data in RDA were tested for significance with an analysis of variance (ANOVA) like permutation test (N = 999). A p-value of < 0.05 was used to identify a significant difference. A total of 705 OTUs were obtained. Core populations were selected based on the following criteria: average relative abundance > 0.5% and occurrence > 50% across both DNA and RNA samples. A phylogenetic tree was constructed using the neighbor-joining method provided in the ARB program^74^.

### Metagenomics and metatranscriptomics

Metagenomic reads were trimmed using Trimmomatic with a quality score of 30, sliding window 6 bp, and a minimum length of 200 bp^75^. To retrieve genomes with high quality, the trimmed reads were processed using a method adapted from a recent study^76^. Briefly, each metagenomic datum was individually assembled, and all data were co-assembled using SPAdes^77^. The assembled contigs were binned using MaxBin2, MetaBAT2, and CONCOCT with default parameters^78–80^. The genomes from the three binning strategies were consolidated using metaWRAP, and the consolidated genomes from the two assembly strategies were combined using dRep^81,82^, with a quality cutoff of > 98% completeness and < 2% contamination using CheckM^83^. A final re-assembly step was performed using SPAdes to further improve the genome quality. Taxonomy classification of MAGs was performed using CAT^84^, and the *Geobacter* populations were extracted to build a phylogenomic tree by including the draft genome of *Ca.* G. eutrophica and publicly available *Geobacter* genomes using Phylophlan^85^. Genes were predicted using Prodigal and annotated using Prokka^86,87^. A Hidden Markov Model database composed of 36 sequences for the conductive type IV *pilA* gene^88^, seven for the genes encoding triheme periplasmic c-type cytochromes (Ppc), and all the genes for the outer membrane c-type cytochrome family (Omc) were manually curated. The *Geobacter* genomes were searched against the database using HMMER for identification of putative genes involved in EET^89,90^. For gene expression analysis, metatranscriptomic reads were trimmed using Trimmomatic with a quality score 30, sliding window 10 bp, and minimum length 100 bp. Ribosomal RNA was removed using SortMeRNA^91^, and the filtered reads were mapped to MAGs using Bowtie 2^92^. The gene expression level was calculated for individual bins as reads per kilobase transcript per million reads (RPKM) using featureCounts^93^.

### Bayesian network analysis

Bayesian networks were constructed as previously described^62^. For the network constructed with 16S rRNA data, abundances of the core population and environmental parameters were combined as a single matrix and normalized to between 0 and 1 using Eq. 1:

1 $$v\_norm_{ij} = \frac{{v_{ij} - min\left( {v_{j} } \right)}}{{max\left( {v_{j} } \right) - min\left( {v_{j} } \right)}}$$

where *v_norm*_*ij*_ is the normalized variable *j* in sample *i*, *v*_*ij*_ is the observed variable *j* in sample *i*, *min(v*_*j*_*)* and *max(v*_*j*_*)* are the minimum and maximum values of variable *j*. Bayesian networks were built with R package “bnlearn” using hill-climbing algorithm, and the parameters of the networks were calculated with Maximum Likelihood parameter estimation method. The networks were examined with leave-one-out cross-validation^94^. Each cross-validation generated a simplified microbial community composed of the core population. Bray–Curtis similarity between the predicted and actual community was calculated and compared with those obtained from a null model. The null model was trained by using average taxa abundance^63^. The correlation between the observed and predicted environmental parameters was performed. The prediction was also validated using relative RMSE, with *y* as the experimental values, *y*_*max*_ as the maximum experimental value, and *t* as the predicted values:

2 $$relative\, RMSE = \frac{{\sqrt {\frac{{\mathop \sum \nolimits_{i = 1}^{n} \left( {y_{i} - t_{i} } \right)^{2} }}{n}} }}{{y_{max} }}$$

After validation, Bayesian networks at the OTU and genus levels were constructed from all datasets. To construct networks with metatranscriptomic data, genes encoding proteins for alcohol metabolism, hydrogen metabolism, EET (putative outer membrane c-type cytochromes and conductive pili), and methanogenesis were selected as the input from dominant fermentative bacteria, *Geobacter* spp. and *Methanobacterium* spp. The model was built following the knowledge about interspecies interaction obtained from community analysis and omics, and the nodes were directed strictly from fermentative bacteria/*Geobacter* over methanogens to CH_4_ using a blacklist function in the bnlearn package. To statistically infer the importance of DIET and IHT, the associated genes were manually silenced (i.e., the expression level was set to 0).

## Supplementary Information

Supplementary Information.

## Data Availability

Sequencing data are available in GenBank under the accession number PRJNA689312.
