# Supplementary material for: Disentangling the syntrophic electron transfer mechanisms of Candidatus geobacter eutrophica through electrochemical stimulation and machine learning
Source: Sci Rep. 2021 Jul 23;11:15140. doi: 10.1038/s41598-021-94628-0 (PMC8302695; doi:10.1038/s41598-021-94628-0)
Supplement: Supplementary file 1 — Supplementary Information. [file 41598_2021_94628_MOESM1_ESM.docx]

**SUPPORTING INFORMATION**

**Disentangling The Syntrophic Electron Transfer Mechanisms of *Candidatus* Geobacter eutrophica through Electrochemical Stimulation and Machine Learning**

Heyang Yuan ^a,b,*^, Xuehao Wang ^a^, Tzu-Yu Lin ^a^, Jinha Kim ^a^, Wen-Tso Liu ^a,*^

^a^ Department of Civil and Environmental Engineering, University of Illinois, Urbana-Champaign, Urbana, IL 61801, United States

^b^ Department of Civil and Environmental Engineering, Temple University, Philadelphia, PA 19122, United States

Type of contribution***: Research Article***

^*^ Corresponding authors.

E-mail: [heyang.yuan@temple.edu](mailto:heyang.yuan@temple.edu)

Supplementary Table S1. Correlation coefficient (R^2^) between the observed and predicted parameters and relative root-mean square error (RMSE) of the prediction. Bayesian networks were trained at the OTU and genus levels with 16S rRNA gene (DNA) or 16S rRNA as the input.

|  | **OTU** | | | | | |  | **Genus** | | | | | |
| --- | --- | --- | --- | --- | --- | --- | --- | --- | --- | --- | --- | --- | --- |
|  | **DNA** | | | **RNA** | | |  | **DNA** | | | **RNA** | | |
|  | R^2^ | RMSE | RMSE std | R^2^ | RMSE | RMSE std |  | R^2^ | RMSE | RMSE std | R^2^ | RMSE | RMSE std |
| COD | 0.87 | 0.08 | 0.07 | 0.78 | 0.11 | 0.08 |  | 0.86 | 0.08 | 0.08 | 0.70 | 0.13 | 0.11 |
| CE | 0.76 | 0.08 | 0.12 | 0.81 | 0.10 | 0.10 |  | 0.64 | 0.12 | 0.13 | 0.67 | 0.12 | 0.12 |
| CH_4_ | 0.86 | 0.09 | 0.07 | 0.94 | 0.07 | 0.07 |  | 0.86 | 0.09 | 0.08 | 0.82 | 0.09 | 0.10 |
| Gas_other | 0.31 | 0.17 | 0.13 | 0.57 | 0.13 | 0.12 |  | 0.31 | 0.16 | 0.15 | 0.42 | 0.14 | 0.14 |
| Acetate | 0.75 | 0.10 | 0.09 | 0.77 | 0.09 | 0.09 |  | 0.71 | 0.10 | 0.10 | 0.79 | 0.08 | 0.09 |
| Propionate | 0.68 | 0.11 | 0.13 | 0.40 | 0.21 | 0.17 |  | 0.73 | 0.11 | 0.10 | 0.71 | 0.11 | 0.11 |
| Butyrate | 0.28 | 0.10 | 0.16 | 0.53 | 0.10 | 0.15 |  | 0.19 | 0.12 | 0.16 | 0.30 | 0.11 | 0.14 |
| Ethanol | 0.89 | 0.05 | 0.08 | 0.92 | 0.04 | 0.07 |  | 0.87 | 0.06 | 0.08 | 0.96 | 0.04 | 0.04 |
| TOC_other | 0.11 | 0.18 | 0.15 | 0.48 | 0.14 | 0.11 |  | 0.40 | 0.14 | 0.12 | 0.29 | 0.16 | 0.12 |
|  |  |  |  |  |  |  |  |  |  |  |  |  |  |
| Mean | 0.61 | 0.11 |  | 0.69 | 0.11 |  |  | 0.62 | 0.11 |  | 0.63 | 0.11 |  |


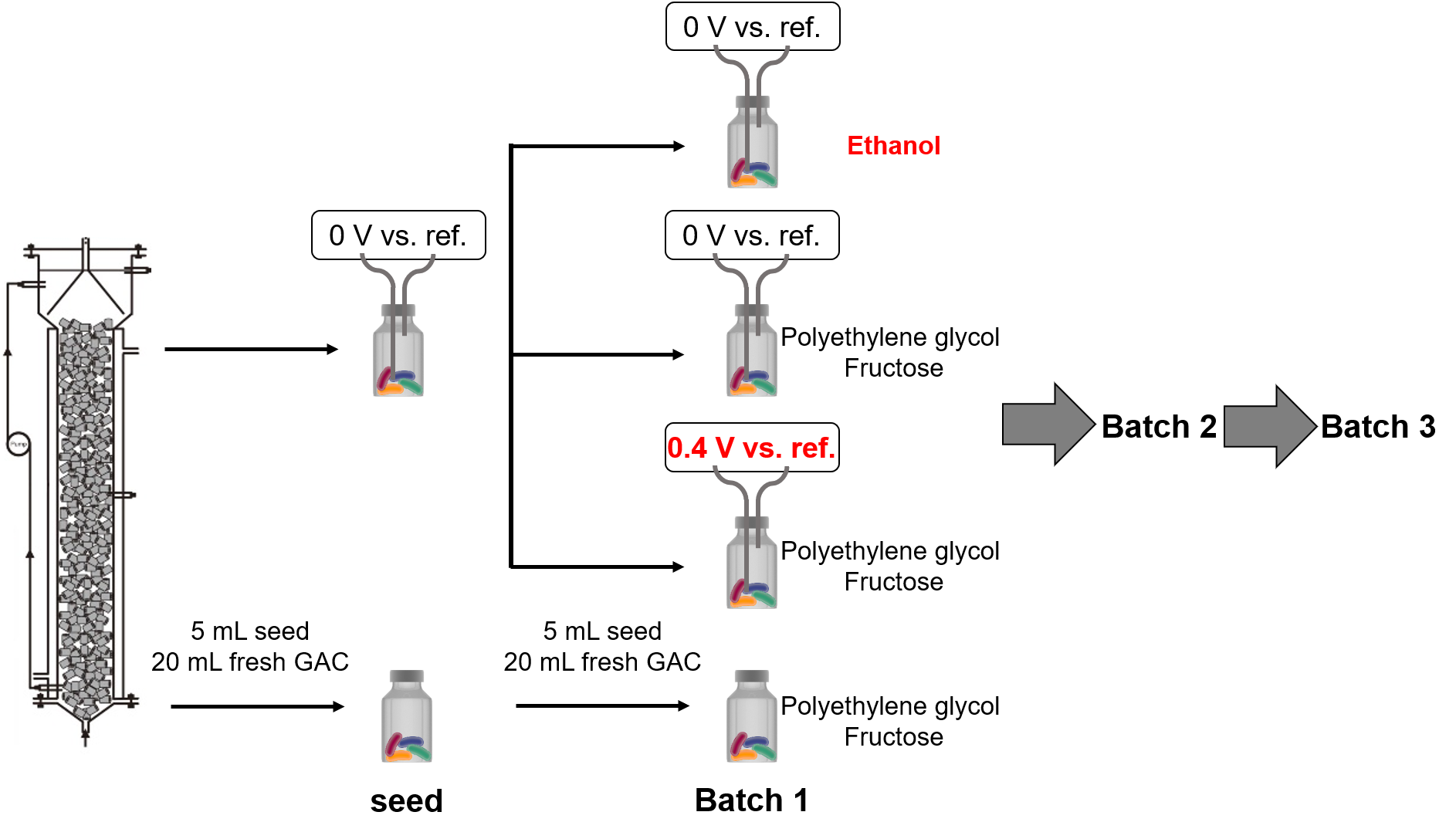


Supplementary Figure S1. Schematic of electrochemical enrichment.


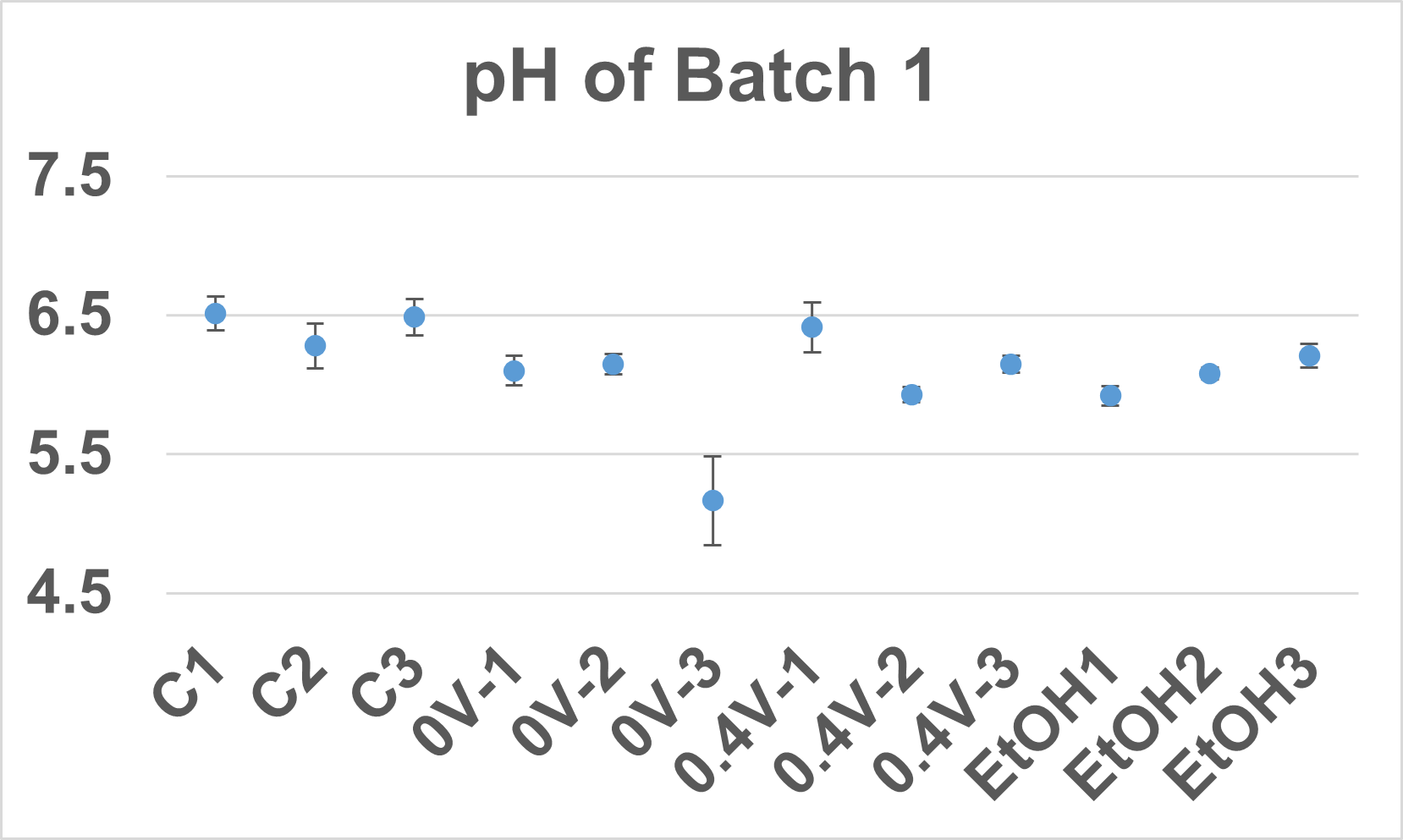


Supplementary Figure S2. pH of the effluent in Batch 1.





Supplementary Figure S3. PCoA based on weighted UniFrac distance was performed with 16S rRNA gene.


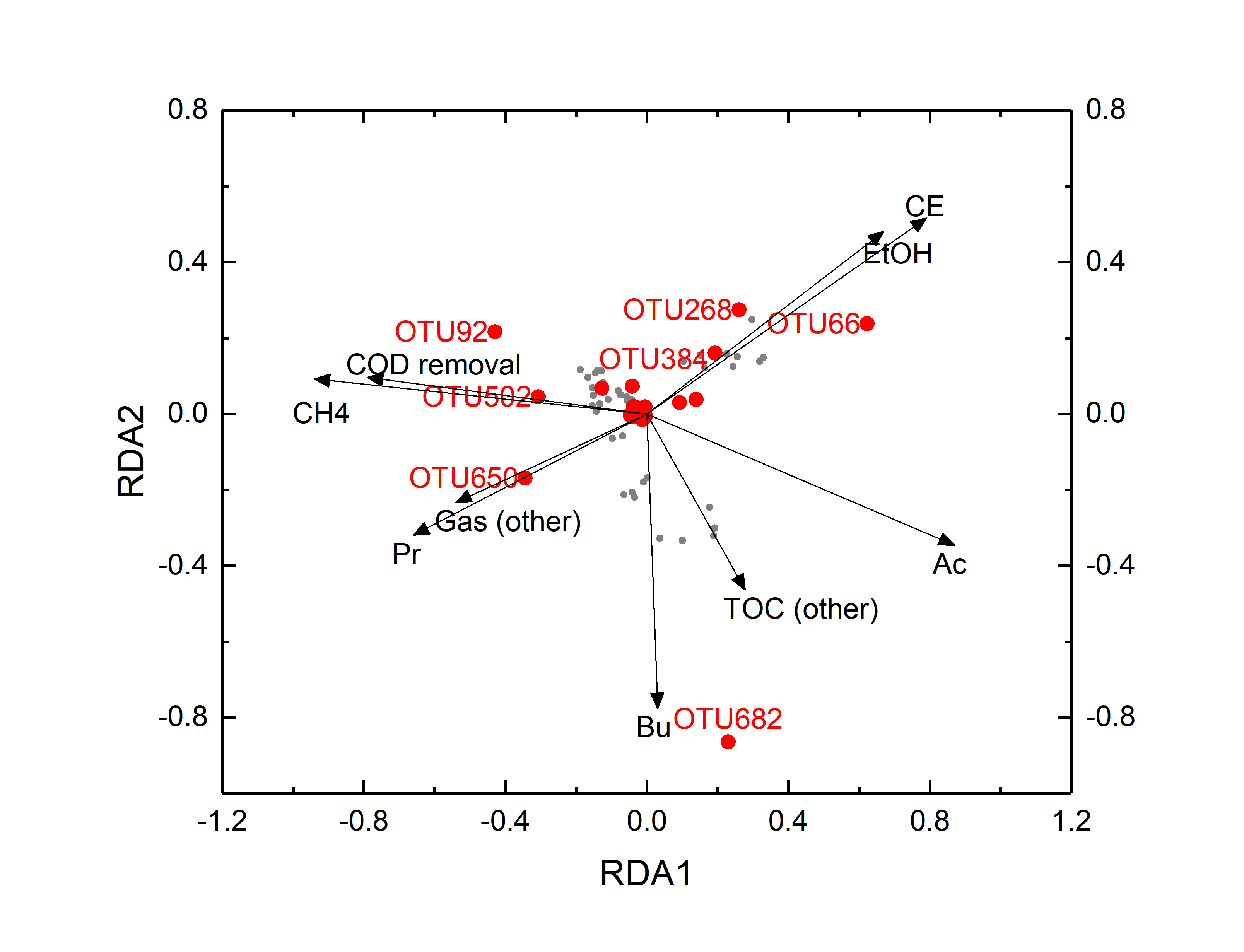


Supplementary Figure S4. RDA was performed with 16S rRNA.


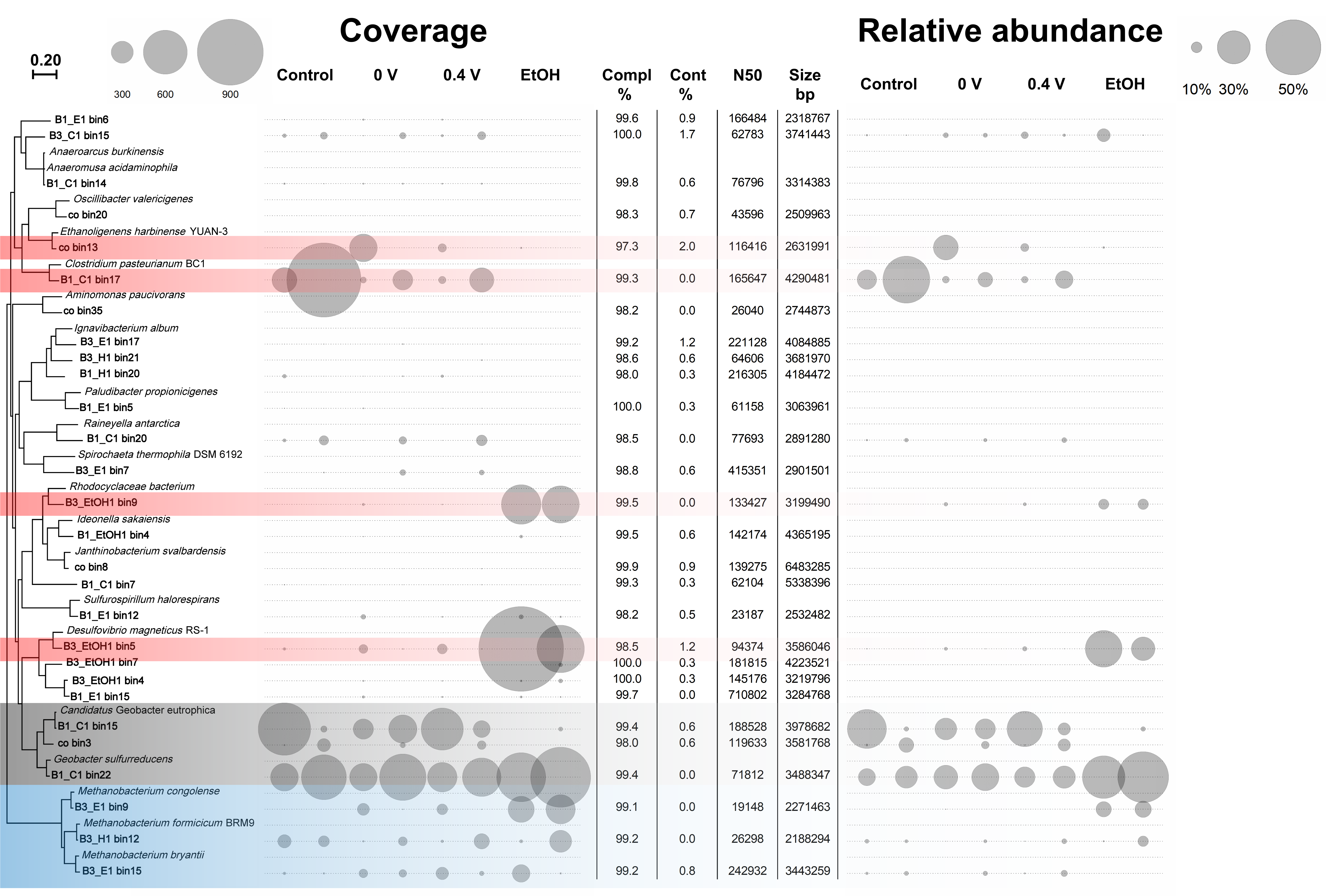


Supplementary Figure S5. Phylogenomic tree, bin coverage, quality, and percentage of reads mapped to 28 MAGs recovered with high quality.


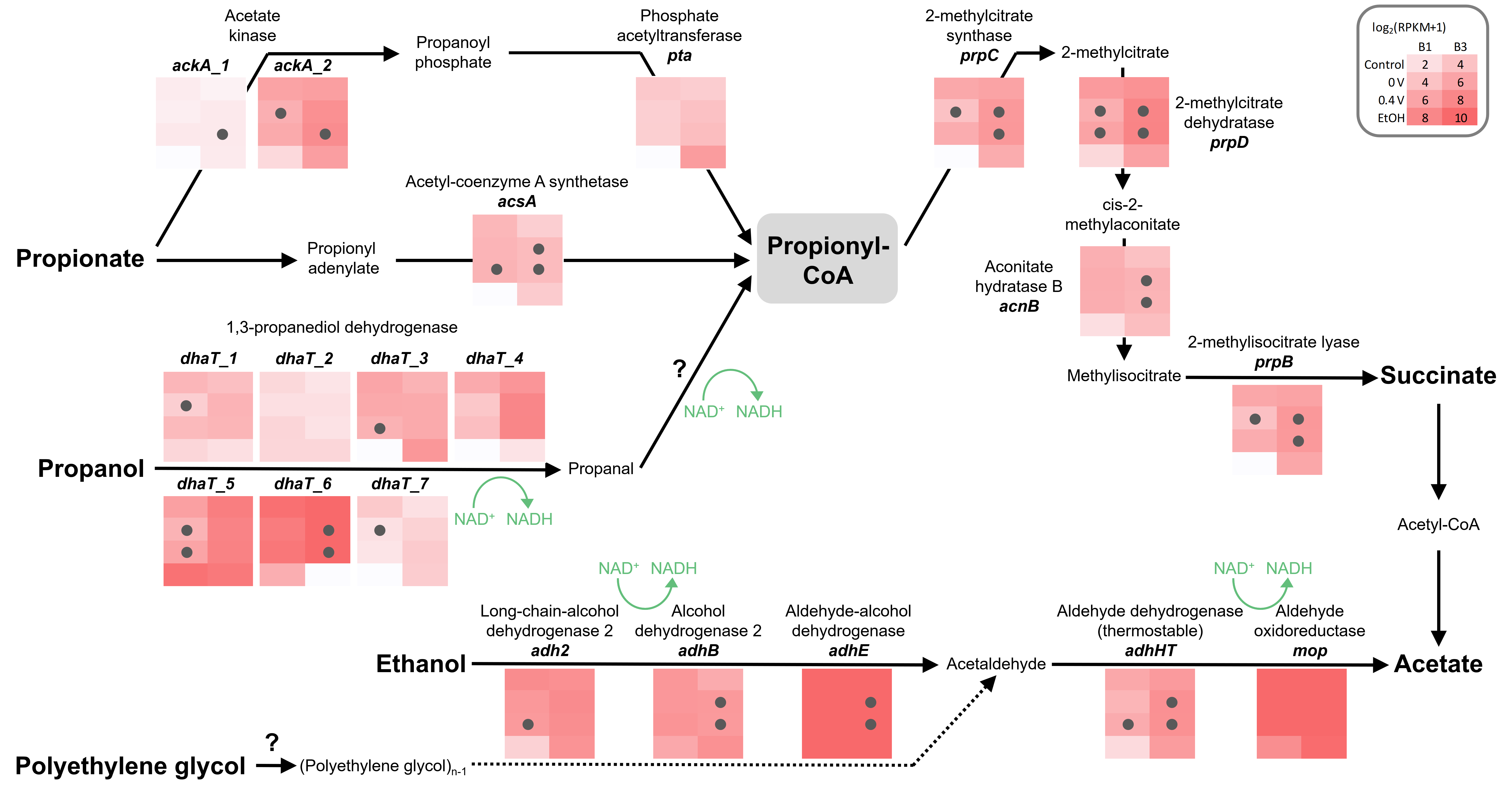


Supplementary Figure S6. Propionate metabolisms of *Geobacter* B1_C1_bin15. The stimulation of propionate metabolism and overall activity by poised electrodes indicated the potential role of propionate as a carbon source during EET and DIET.


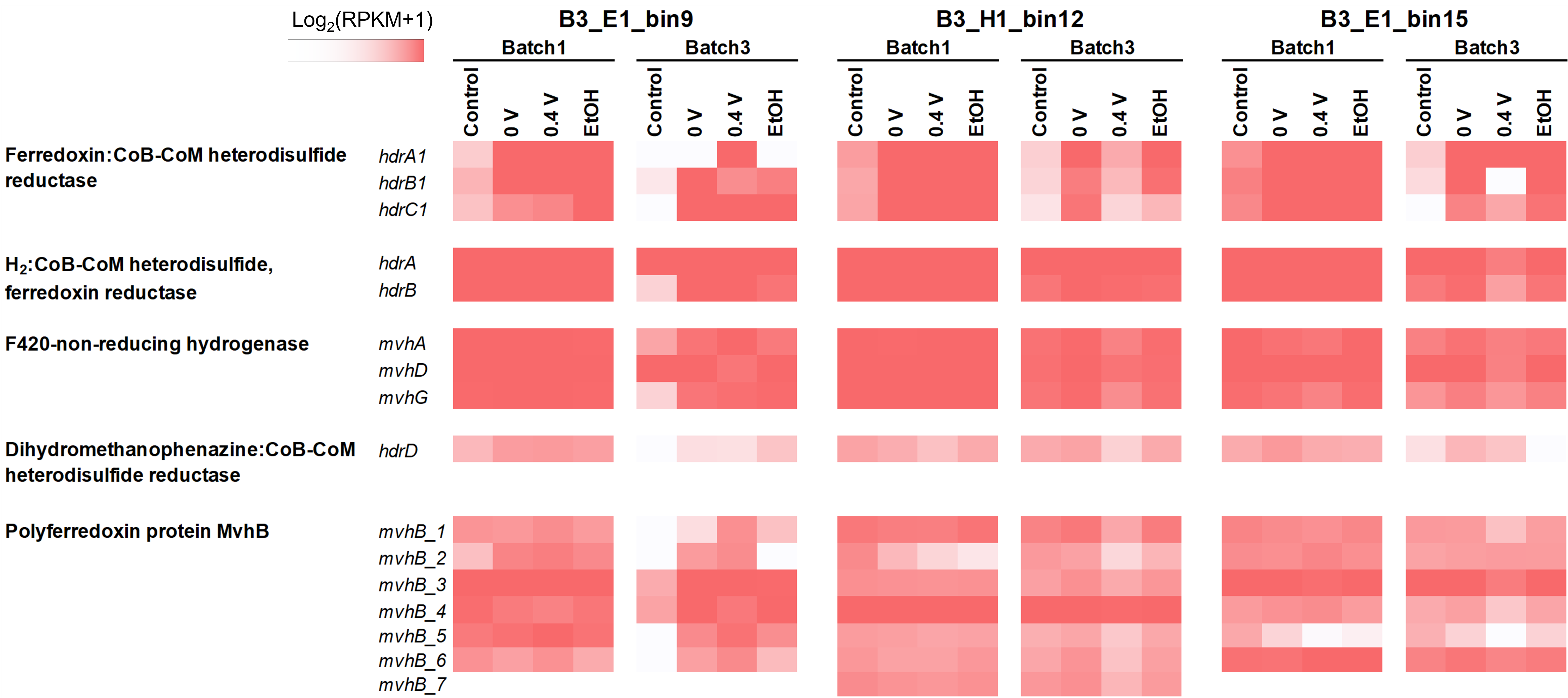


Supplementary Figure S7. Expression of the genes for the enzymes involved in coenzyme B and M recycling and polyferredoxin MvhB in three abundant *Methanobacterium* spp. Heatmap is created using Microsoft Excel (Microsoft 365).





Supplementary Figure S8. Bray-Curtis similarity between the observed and predicted communities obtained from Bayesian network and a null model constructed using DNA and RNA data.
